# Tick infestation in spur-thighed tortoise population: a pilot study for unraveling epidemiological patterns and demographic consequences

**DOI:** 10.1007/s10493-023-00863-7

**Published:** 2023-11-16

**Authors:** Amalia Segura, Marta Rafael, Rita Vaz-Rodrigues, Oscar Rodríguez, Christian Gortázar, José de la Fuente

**Affiliations:** 1 BP 30, Sidi Allal el Bahraoui, 15250 Morocco; 2 SaBio, Instituto de Investigación en Recursos Cinegéticos (IREC), Consejo Superior de Investigaciones Científicas (CSIC), Universidad de Castilla-La Mancha (UCLM)-Junta de Comunidades de Castilla-La Mancha (JCCM), Ronda de Toledo 12, Ciudad Real, 13005 Spain; 3 Center for Veterinary Health Sciences, Department of Veterinary Pathobiology, Oklahoma State University, Stillwater, OK 74078 USA

**Keywords:** *Hyalomma aegyptium*, Surveillance, Tick-borne infectious agents, Tortoise, *Testudo graeca*

## Abstract

Ectoparasites, such as ticks, modulate host population dynamics by impacting demographic traits. They transmit infectious agents among their hosts, posing a critical threat to animal and public health. This study aimed to characterize and analyze the *Hyalomma aegyptium* infestation on one of its main hosts, the spur-thighed tortoise, its effects on demographic traits, and to determine the diversity of infectious agents present in both ticks and tortoises in the Maamora forest (northwestern Morocco). Our results show that 100% of the tortoises were parasitized by adult ticks in spring, an infestation intensity of 4 ticks/tortoise (5.1 and 3.6 ticks/tortoise in males and females, respectively; 4.2 and 3.3 ticks/tortoise in gravid and non-gravid females, respectively) and an abundance ranging from 1 to 12. Although without significant differences, male tortoises had higher tick abundances than females. The interaction of tortoise sex and body condition was significantly related to tick abundance, male body condition decreased with higher tick abundance in contrast to females. Nevertheless, the interaction of body condition and reproductive stage of females was not significantly related to tick abundance. Gravid females were significantly associated with tick abundance, showing a slightly higher infestation than non-gravid females. Molecular analysis of pooled tick samples revealed the presence of *Ehrlichia ewingii*, *Candidatus* Midichloria mitochondrii, and *Rickettsia africae*, with a minimum infection rate of 0.61 to 1.84%. However, blood sample analysis of the tortoises was infectious agent-free, pinpointing a lack of significant health problems. Given the possible effect on the transmission of zoonotic diseases by spur-thighed tortoises associated with their frequent collection as pets, it should be surveyed to control possible human health problems. In conservation terms, as a long-lived species, the role of tick infestation in demographic traits might be included in the management and conservation programs of spur-thighed tortoises.

**Supplementary Information:**

The online version contains supplementary material available at 10.1007/s10493-023-00863-7.

## Introduction

Ectoparasites may modulate host population dynamics by influencing natural selection (Fitze et al. 2004; Bull and Burzacott 2006). Long interaction between hosts and ectoparasites impacts host population structure and size, affecting defence effectiveness and resulting in most of the cases in adaptation and co-evolution (Hwang and Kuang 2003; Esser et al. 2019). Tortoises from the *Testudo* genus have been deeply documented as hosts of tick species of the *Hyalomma* genus, such as *Hyalomma aegyptium* L. (Hoogstraal and Kaiser 1960; Široký et al. 2006), affecting those ectoparasites their life-history traits. Particularly high is the encounter rate of *H. aegyptium* with spur-thighed tortoise *Testudo graeca* in Morocco, Tunisia, Turkey and Algeria (Gharbi et al. 2015; Tiar et al. 2016; Segura et al. 2019; Najjar et al. 2020), and to a lesser extend with Marginated tortoise *Testudo marginata* in Greece (Široký et al. 2006), Horsfield´s tortoise *Testudo horsfieldii* in Iran (Javanbakht et al. 2015), and Hermann´s tortoise *Testudo hermanni* in Albania (Hoogstraal, 1956; Široký et al. 2006; Bizhga et al. 2022). The endured contact between *H. aegyptium* and *Testudo* may depend on a complex interplay of factors involving host demographic factors such as sex, reproductive stage or population density, host-parasite factors including encounter, compatibility and recognition strategies (Hoberg and Brooks 2008) and abiotic factors including elevation, temperature, rainfall and humidity (Cumming 2002; Javanbakht et al. 2015). In particular, the effect of tick parasitism is often higher in male tortoises than in females (Segura et al. 2019; Laghzaoui et al. 2022; but see Tiar et al. 2016), representing either differences in exposure or susceptibility to ticks, such as male-specific behaviour in breeding time by differential habitat use (Robbins et al. 1998). Male parasitism might result in an extra biological cost if physiological aspects such as body condition are affected (Segura et al. 2019). The effect of parasitism in the reproduction of tortoise females may influence resource allocation trade-offs, reducing or increasing reproductive output according to different strategies (e.g., Lockley et al. 2020). Therefore, female reproductive success might be compromised as a direct consequence of resource exploitation by parasites. Whereas small (young) infected females could use a bet-hedging strategy in favour of lifetime reproductive success, older infected females could adopt a terminal investment strategy (e.g., Lockley et al. 2020). Additional external factors, such as the limitation of food resources, will favour resource allocation from current reproduction to survival (and future reproduction) until the infection has passed (e.g., Hurd 2001; Pollock et al. 2012).

Adults of *H. aegyptium* feed almost exclusively on tortoises of the genus *Testudo*. However, rare cases in other hosts, such as hares and hedgehogs, have been reported (Hoogstraal and Kaiser 1960; Gazyağci et al. 2010). Larvae and nymphs are less host-specific and feed on a variety of vertebrates (Estrada-Peña et al., 2017), including domestic animals (dogs, cattle, horses, or pigs; Aydin 2000), wild animals (lizards, birds, hedgehogs, rodents, or camels; Kar et al. 2011; Široký et al. 2011; Apanaskevich and Oliver 2014), and humans (Vatansever et al. 2008; Bursali et al., 2010). Ticks are considered the second vector of human diseases and are both vectors and reservoirs of infectious agents, harbouring bacterial, viral, and protozoan microorganisms (de la Fuente et al. 2017). The multitude of hosts affected by *H. aegyptium* poses a major concern as various dissemination scenarios may occur, leading to epidemiological consequences. Indeed, several infectious agents have been detected in *H. aegyptium* collected from spur-thighed tortoise, such as *Rickettsia* spp., *Ehrlichia* spp., *Anaplasma* spp., *Coxiella burnetii*, Crimean-Congo haemorrhagic fever virus (CCHFV) or *Hemolivia mauritanica* (Tiar et al. 2010; Bursali et al., 2011; Paștiu et al. 2012; Kautman et al. 2016; Barradas et al. 2019, 2020; Manoj et al. 2021; Mumcuoglu et al. 2022; Rjeibi et al. 2022). Particularly in Morocco, the presence of *H. mauritanica*, *Ehrlichia* spp., Midichloria mitochondrii, *Wolbachia* spp., relapsing fever borreliae, *Francisella* spp., and *Rickettsia* spp. has been reported from spur-thighed tortoise infested by *H. aegyptium* (e.g., Harris et al. 2013; Norte et al. 2021).

In our study, we examined the presence of infectious agents in both *H. aegyptium* ticks and spur-thighed tortoises and the role of sex and female reproductive stage in tortoises as drivers of tick infestation in the host species. Spur-thighed tortoise have been red-listed as ‘vulnerable’ by the International Union for Conservation of Nature (IUCN 1996; Rhodin et al. 2021) and one of their main threats through their distribution is the collection and trade as pets (Pérez et al. 2004; Tiar et al. 2019; Segura et al. 2020). We selected a population located in the Maamora forest, a cork oak forest located in northern Morocco that is characterized as highly humid, when comparing with other areas of the tortoise distribution range, and considered close to the optimum niche of the tortoise distribution (Anadón et al. 2012). The population has been previously studied in 2018 in a private reserve where there is no pet trade and the undergrowth is well preserved (Segura et al. 2020). The study revealed high prevalence and moderate intensity of tick parasitism, and the influence of tick infestation on tortoise age, sex, body condition and population density (Segura et al. 2019). Indeed, this spur-thighed tortoise population has been recognized as one of the densest documented to date (Segura and Acevedo 2019). However, the epidemiological status of the tortoise community present in the Maamora forest is unknown, even though several demographic studies had discussed the different drivers of this tortoise population (Segura and Acevedo 2019; Segura et al. 2019, 2021). The high collection and trade of the species in this forest (Segura et al. 2020) pinpoint to the potential transmission of zoonotic pathogen agents. This study aims to (i) determine adult parasite prevalence, intensity and abundance in tortoises, (ii) analyse the role of tortoise sex, tortoise female reproduction stage and the interaction of both factors with the body condition as drivers of tick parasitism in the species, and (iii) identify and phylogenetically characterize tick-borne infectious agents, including *Anaplasma* spp., *Babesia* spp., *C. burnetii*, *Ehrlichia* spp., *Hepatozoon* spp. / *H. mauritanica*, *Rickettsia* spp., *Borrelia* spp., and CCHFV, in both *H. aegyptium* ticks and the spur-thighed tortoise. This study will contribute to the design of appropriate management and conservation plans and emphasizes the importance of surveillance and epidemiological profiling of both vectors and hosts.

## Materials and methods

### Study site

The study was conducted in an area of low-elevation sandy soil (72–185 m above sea level) in the Maamora forest (Northwest Morocco; 34°02′54.19′′ N, 6°27′19.24′′ W, Grou-Bouregreg basin). The study area was located on the Mediterranean bioclimatic floor, with hot and dry summers, and the annual range of average rainfall was 300–500 mm and the mean annual temperature 22º C. Maamora forest is dominated by cork oak trees *Quercus suber*, scattered endemic wild pear *Pyrus mamorensis*, wild olive *Olea europaea*, green olive *Phyllirea latifolia*, and mastic *Pistacia lentiscus*, and a sparse understory of bush and shrub species such as Mediterranean broom *Genista linifolia*, *Cytisus arboreus, Stauracanthus genistoides*, dwarf palm *Chamaerops humilis*, French lavender *Lavandula stoechas*, sage-leaved rockrose *Cistus salviifolius*, *Halimium halimifolium*, and *Thymelaea lythroides*. The study took place on a private reserve (3000 ha) characterized by well-represented undergrowth (e.g., species richness and cover) when compared with other unprotected sites in Maamora (highly overgrazed by livestock; Said et al. 2014).

### Sampling

**Fig. 1:**
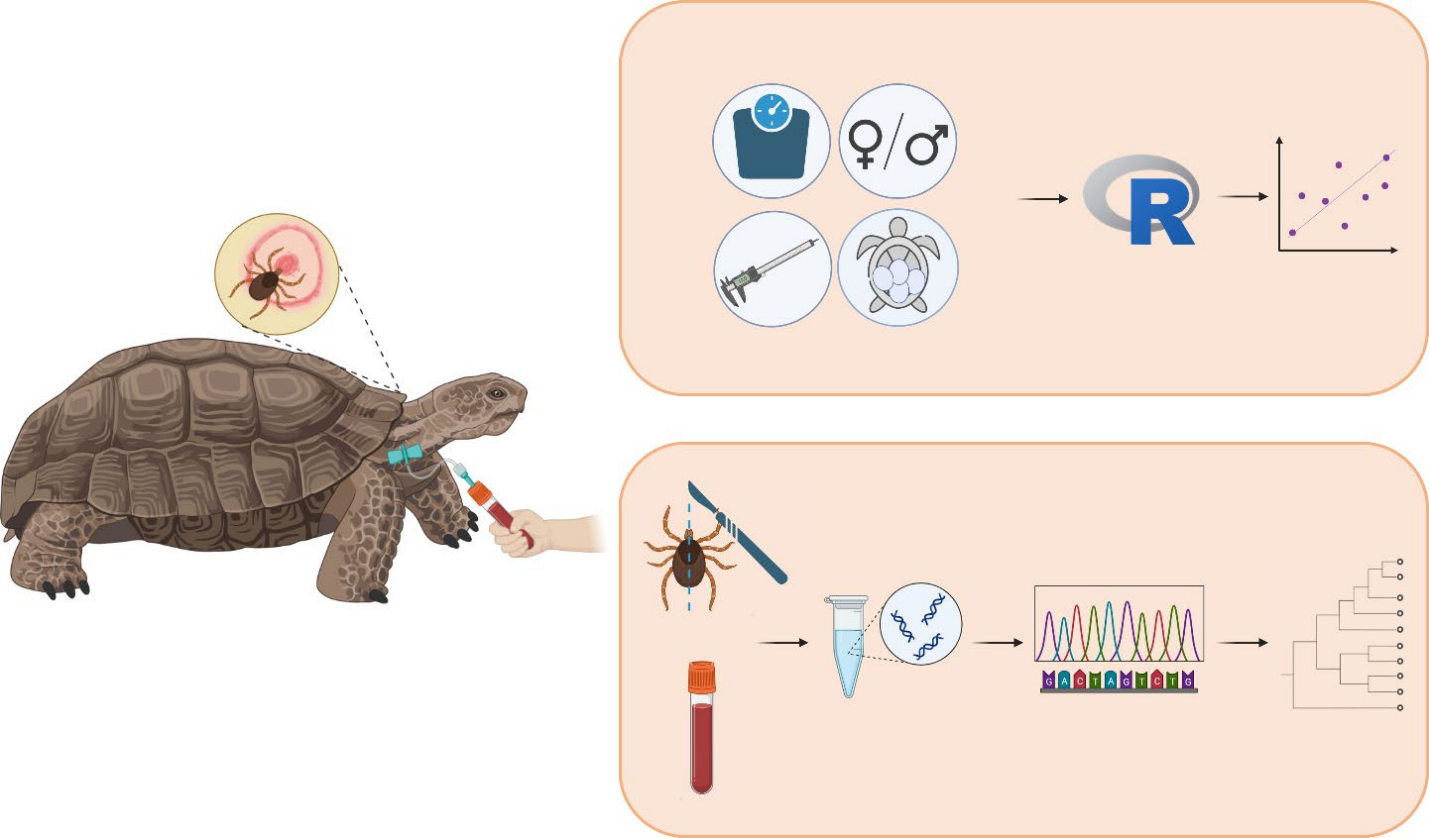
Methodological flowchart. Individual characteristics were recorded such as weight, sex, body size measures and quantification of eggs. The relationship between the variables was performed using general linear models and linear models with the R software. In addition, DNA was extracted from ticks and blood samples collected from the tortoises. Positive samples of the pathogens analysed were sequenced, and phylogenetic trees were generated

Tortoises were captured by hand between April and May 2022 (Table S1) following approved ethical wildlife capture and management protocols. Each individual encountered was sexed, the body mass was determined using a precise balance (± 1 g), and the body size was measured (± 1 mm) as the straight anteroposterior distance between the nuchal and supracaudal scutes using a calliper (carapace length, CL). Collection and tick extraction were carried out within a private initiative for the conservation of *T. graeca* in Maamora Forest. All ticks attached to the tortoise body were counted in the field, and a representative subsample was collected for analysis of infectious agents. The removed ticks were identified at the species level with DNA barcoding of mitochondrial genes. Blood was collected from the subcarapacial plexus using a 1-mL syringe. For determining the female reproductive stage, females were radiographed dorsoventrally with a portable X-ray at 60 kV (20 mA) at a distance of 1 m, according to Gibbons and Greene (1979). The radiography allowed the identification of gravid females and assessed the clutch size. Figure 1 represents the methods employed in this article. All tortoises were released immediately after measurements and sample collections at the capture site.

Three parasitological indicators were calculated: (1) infestation prevalence, by dividing the number of infested tortoises by the number of examined tortoises and multiplying it by 100, (2) mean infestation intensity, by dividing the number of ticks by the number of infested tortoises, and (3) tick abundance, by dividing the number of ticks by the number of examined tortoises. The tortoise body condition (BC), which represents the body mass adjusted to the body size (Nagy and Medica 1986), was determined by calculating residual values through a linear regression analysis (all individuals pooled). In this analysis, the natural logarithm (ln) of body mass was used as the dependent variable, whereas ln CL was used as the independent variable. The individual body-condition index measures the extent of mass deviation compared to the expected values based on the animal’s size, which can change with age, stage of reproduction, drought and disease.

Both the ticks and the blood of the tortoises were stored at -25 °C in tubes with RNAlater and sodium heparin, respectively, for further analysis.

### Tick DNA/RNA isolation and PCR infectious agents analysis

Nucleic acid extraction was accomplished from individual tick samples and tick pools (mean of 3,196 ticks/pool, ranging from 1 to 8 ticks). The pools were designed randomly, according to the number of ticks collected in the field. DNA and RNA were extracted from the internal tissues of ticks, discarding the external cuticle, and using TRI Reagent (Sigma-Aldrich, St. Louis, USA), following the manufacturer’s instructions. The concentration (ng/µL) and purity of samples were evaluated using a Nanodrop One spectrophotometer (Thermo Scientific, Waltham, USA), through the quantification of the nucleic acids at an optical density of 260 nm (OD260) and the ratio of absorbance at 260/280 nm. The quality of the extraction protocol and confirmation of tick species were appraised by the amplification of the mitochondrial *16S ribosomal DNA* (*16S rDNA*) gene and the *cytochrome oxidase subunit I* (*COI*) *gene* of four individual ticks (Table 1). All samples were tested using conventional polymerase chain reaction (PCR) aimed at detecting the presence of *Anaplasma* spp., *Babesia* spp., *C. burnetii*, *Ehrlichia* spp., *Hepatozoon* spp. / *H. mauritanica*, or *Rickettsia* spp., a nested PCR for the detection of *Borrelia* spp., and a nested reverse transcription (RT)-PCR for the identification of the CCHFV. Table 1 provides information on the specific targeted regions for each PCR assay, the used protocol, and primers.

**Table 1:** Primers and PCR protocols according to the pathogen analysed

Pathogen and target gene	Sequence 5’-3’ (F: Forward / R: Reverse)	Fragment (bp)	Annealing (ºC)	Reference
*16S rDNA*	F: CCGGTCTGAACTCAGATCAAGT R: CTGCTCAATGATTTTTTAAATTGCTGTGG	460	48	Rodríguez et al. 2022
*COI*	F: GGTCAACAAATCATAAAGATATTGG R: TAAACTTCAGGGTGACCAAAAATCA	650	50	Coimbra-Dores et al. 2018
*Anaplasma* spp. (*16S rRNA*)	F: CAGAGTTTGATCCTGGCTCAGAACG R: GAGTTTGCCGGGACTTCTTCTGTA	421	42	Moraga Fernández et al. 2022
*Anaplasma* spp. (*msp5*)	F: GCATAGCCTCCGCGTCTTTC R: TCCTCGCCTTGGCCCTCAGA	456	54	Moraga Fernández et al. 2022
*Anaplasma* spp. (*msp4*)	F: CGGATCCTTAGCTGAACAGGAATCTTGC R: GGGAGCTCCTATGAATTACAGAGAATTGTTTAC	849	60	Moraga Fernández et al. 2022
*Babesia* spp. (*18S rRNA*)	F: AAT ACC CAA TCC TGA CAC AGG G R: TTA AAT ACG AAT GCC CCC ACC	408	58	Barradas et al. 2020
*Borrelia burgdorferi* sensu lato (*flagellin*)	F1: GCATCACTTTCAGGGTCTCA R1: TGGGGAACTTGATTAGCCTG F2: CTTTAAGAGTTCATGTTGGAG R2: TCATTGCCATTGCAGATTGT	390	55 and 58	Norte et al. 2021
*Coxiella burnetii* (*IS111a*)	F: CAAGAATGATCGTAACGATGCGC R: CTCGTAACACCAATCGCTTCG	349	63	Rjeibi et al. 2022
Crimean-Congo Haemorrhagic Fever vírus (CCHFV S segment)	F1: TTGTGTTCCAGATGGCCAGC R1: CTTAAGGCTGCCGTGTTTGC F2: GAAGCAACCAARTTCTGTGC R2: AAACCTATGTCCTTCCTCC	211	60 and 57	Moraga-Fernández et al. 2021
*Ehrlichia* spp. (*16S rRNA*)	F: GGTACCYACAGAAGAAGTCC R: TAGCACTCATCGTTTACAGC	345	54	Barradas et al. 2020; Gal et al. 2008
*Hepatozoon* spp. / Hemolivia mauritanica (*18S rRNA*)	F: GTTTCTGACCTATCAGCTTTCGACG R: CAAATCTAAGAATTTCACCTCTGAC	600	60	Norte et al. 2021; Ujvari et al. 2004
*Rickettsia* spp. (*16S rRNA*)	F: AGAGTTTGATCCTGGCTCAG R: AACGTCATTATCTTCCTTGC	416	54	Rodríguez et al. 2022
*Rickettsia* spp. (*ompA*)	F: ATGGCGAATATTTCTCCAAAA R: AGTGCAGCATTCGCTCCCCCT	630	54	Moraga-Fernández et al. 2019
*Rickettsia* spp. (*ompB*)	F: GGGTGCTGCTACACAGCAGAA R: CCGTCACCGATATTAATTGCC	618	53	Moraga-Fernández et al. 2019

The PCR reactions were performed in a 25 µL volume, including 12.5 µL of PCR Master Mix 2x (Promega, Madison, WI, USA), 1 µL of each primer (10 µM working solution), 9 µL of RNase-free water (Thermo Scientific), and 1.5 µL of DNA sample. For the nested RT-PCR assessment of CCHFV, the commercial kit Access RT-PCR System (Promega, Fitchburg, WI, USA) was used according to the manufacturer’s instructions. The PCRs were conducted in a C1000 touch PCR thermal cycler (Bio-Rad, Hercules, CA, USA), with the specific PCR fragments visualized in 1.5% agarose gel stained with GelRed (Biotium, Fremont, CA, USA) under UV transillumination.

### Sequencing and phylogenetic analysis

Presumed positive samples were purified and sequenced using the Sanger method at Secugen (Madrid, Spain). Sequences were edited with the Chromas software v.2.6.6., and homology analysis was conducted using the National Center for Biotechnology Information (NCBI) database, employing the Basic Local Alignment Search Tool (BLAST). The *16S rDNA* sequence was deposited in GenBank under the accession number OQ295899. The *COI* partial sequences obtained in this study were attributed the accession numbers OQ320497 and OQ556797. The *16S rRNA* partial sequences of *Ehrlichia* identified in this study were ascribed the accession numbers OQ9931657, OQ991496, OQ991497 and OQ996270. The *outer membrane protein A* [*ompA*] partial sequence of *Rickettsia* was submitted to Genbank and assigned the accession number OR003919. Multiple sequence alignment was carried out using the Multiple Sequence Comparison by Log-Expectation (MUSCLE) algorithm. Phylogenetic analysis was performed in MEGA software v.11.0.13. Corrected Akaike Information Criterion (cAIC) was used to select the best-fit model, and a phylogenetic tree for positive infectious agents was generated using maximum likelihood and Neighbor-Joining methods. To ensure the reliability of produced trees, 1000 bootstrap replicates were implemented.

### Blood nucleic acid isolation and PCR infectious agent analysis

Blood DNA was extracted from tortoises with suspected infectious agents present in tick samples using the DNeasy Blood & Tissue Kit (Qiagen, Hilden, Germany) and following the manufacturer’s instructions. The samples were tested using conventional PCR against *Anaplasma* spp., *Ehrlichia* spp. and *Rickettsia* spp. (Table 1). The PCR protocol followed the same indications as the one described for the tick infectious agents research.

### Statistical analysis

χ^2^ tests were used to assess differences in infestation intensity between tortoise sexes and between gravid and non-gravid tortoise females. Two generalized linear models (GLM) with a Poisson distribution and logarithmic link function were performed with the R v.4.3.1 (2023) software, to analyse the relationship between tick infestation rate (tick abundance) and (i) the tortoise sex and the interaction of body condition with sex, and (ii) female reproductive stage (gravid/non-gravid females) and the interaction of body condition with reproductive stage. For all analyses, statistical significance was declared at α = 0.05 (confidence level of 95%).

## Results

### Tick infestation rate and tortoise demographic traits

In total 520 ticks (mostly adults with the exception of four nymphs) were counted on the 130 tortoises captured (98 females, 32 males). Overall, the infestation prevalence was 100% with all the tortoises parasitized by ticks, and the mean (± 95% confidence interval) infestation intensity was 4 ± 0.42 ticks/tortoise. Tick abundance ranged from 1 to 12 ticks/tortoise.

**Fig. 2:**
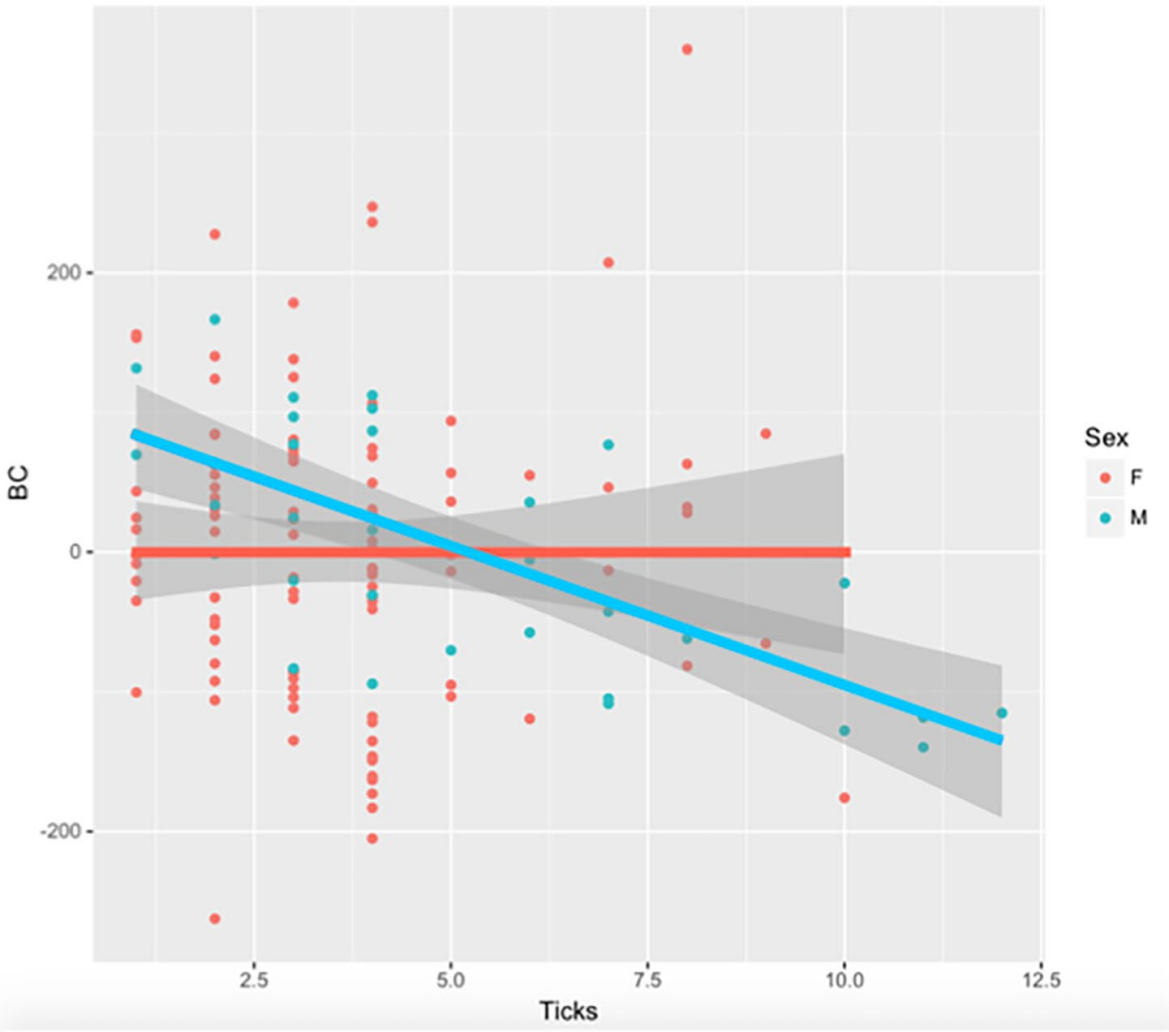
Number of ticks encountered categorized by sex and according to body condition (BC). Female data are represented in red, male data in blue

Males presented higher infestation intensity (5.3 ± 1.11 ticks/tortoise) than females (3.6 ± 0.40 ticks/tortoise) but the differences between them were not significant (χ^2^ = 2.4, d.f. = 1, *P* = 0.1). The model for determining the infestation rate effect on sex and body condition showed a significant relation of sex and a significant interaction between body condition and sex. Males had higher tick abundances, and tick abundance decreased in males in relation to their body condition (Table 2; Fig. 2).

**Table 2:** Statistical parameters of the generalized linear model (GLM) carried out to determine tick abundance variation in relation to the tortoise sex and the interaction of body condition and sex in tortoises

Model predictors	Estimate	SE	t	*P*
(Intercept)	1.287e + 00	5.307e-02	24.252	< 0.01
Body condition	-2.617e-05	4.945e-04	-0.053	0.96
Sex^1^				
Males	2.620e-01	1.003e-01	2.613	< 0.01
Body condition × males	-4.978e-03	1.083e-03	-4.597	< 0.01

^1^Class reference for the categorical variable sex is ‘female’

Gravid females (34%; 1–5 eggs) presented higher mean infestation intensity than non-gravid females (4.2 vs. 3.3 ticks/tortoise, n = 33 and 65, respectively) but the differences between them were not significant (χ^2^ = 1.08, d.f. = 1, *P* = 0.29). The model showed a significant relation between tick abundance and the reproductive stage of the females, gravid females with higher tick abundance than non-gravid females. In addition, it showed a lack of significance in the interaction between body condition and the reproductive stage of the females (Table 3).

**Table 3:** Statistical parameters of the generalized linear model (GLM) carried out to determine tick abundance variation in relation to the reproductive stage (gravid and non-gravid females) and the interaction of body condition and reproductive stage

Model predictors	Estimate	SE	t	*P*
(Intercept)	1.448	0.084	17.108	< 0.01
Reproductive stage				
Non-gravid^1^	-0.251	0.108	-2.316	< 0.01
Body condition	-0.0004	0.0009	-0.478	0.63
Body condition × non-gravid	0.0004	0.001	0.449	0.65

^1^Class reference for the categorical variable sex is ‘gravid’

### PCR analysis

**Fig. 3:**
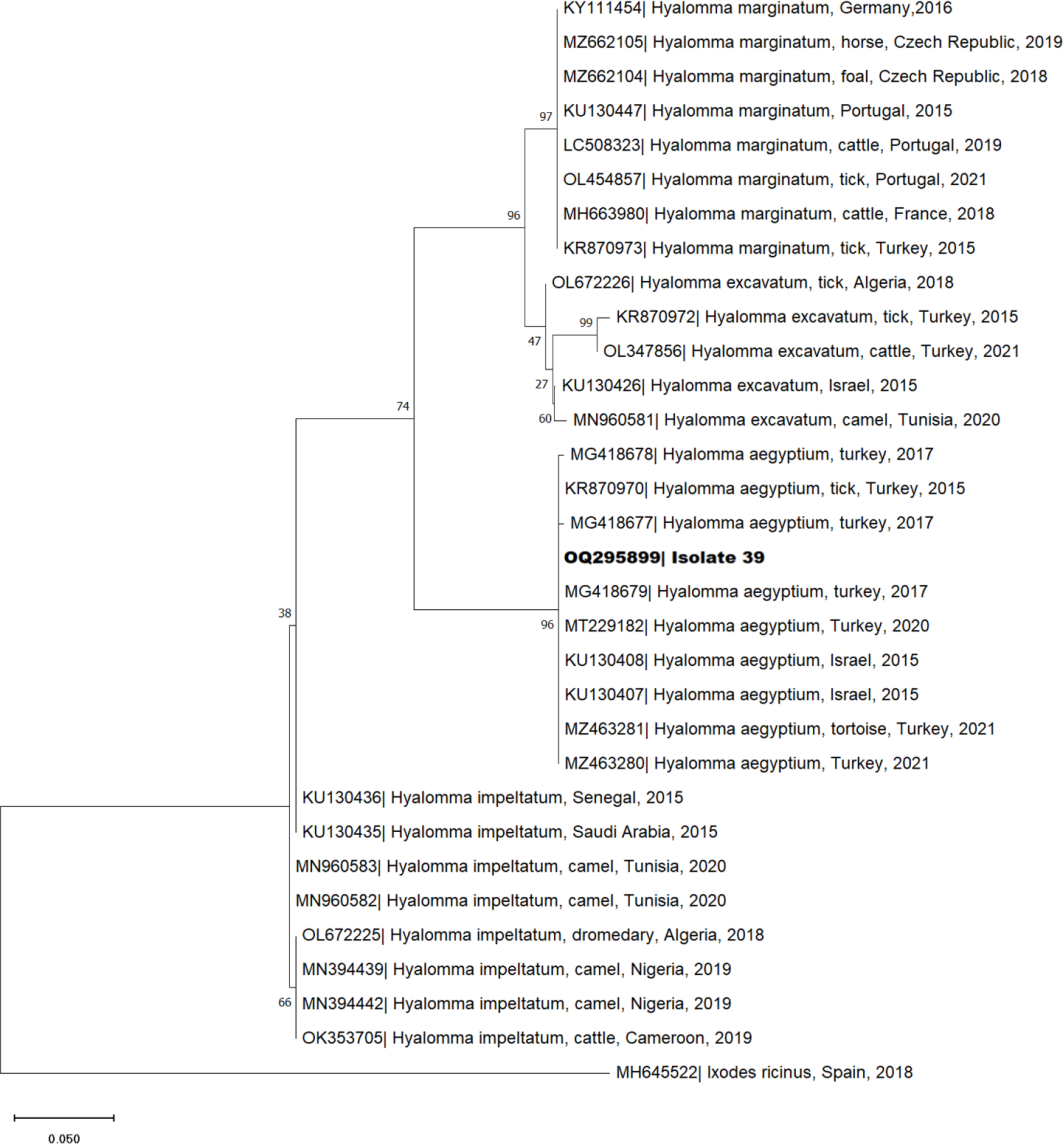
Phylogenetic tree of mitochondrial *16S rDNA* sequences of *Hyalomma aegyptium* isolated from spur-thighed tortoise (*Testudo graeca*), Morocco. The analysis was obtained based on the Neighbor-joining method with Tamura-3-parameter with a discrete Gamma distribution model. The characterized species in this study are represented in bold. Sequence names include the GenBank accession number, organism name, host (if mentioned), country of origin and year of collection or submission. The reliability of internal branches was assessed using the bootstrapping method with 1000 replicates

**Fig. 4:**
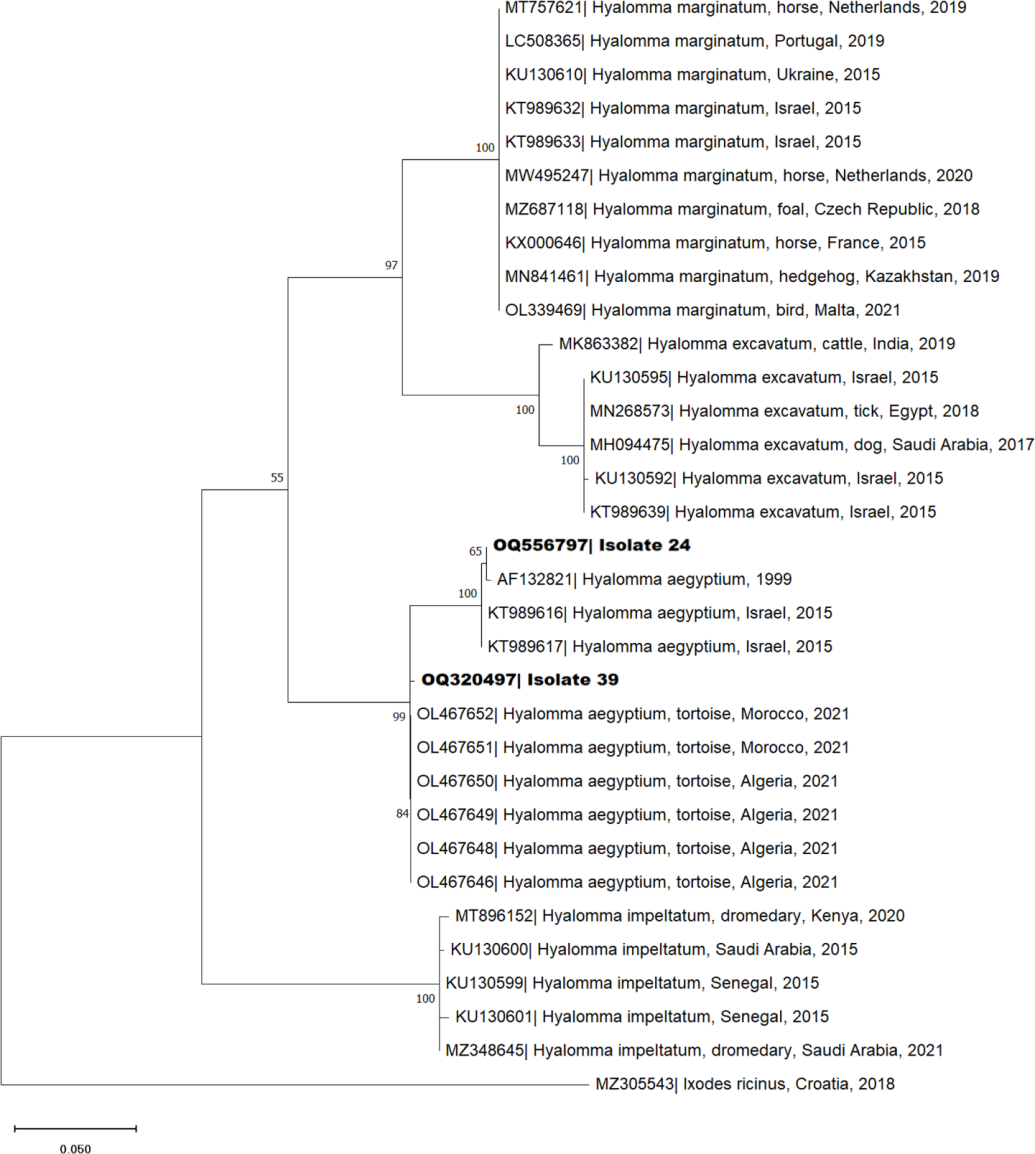
Phylogenetic tree of *COI* sequences of *Hyalomma aegyptium* isolated from spur-thighed tortoise (*Testudo graeca*), Morocco. See Fig. 3 for details of the analysis

A sample of 163 ticks (156 males, six females, and one nymph) was used for DNA extraction and analysis. All 163 ticks were confirmed as *H. aegyptium* by barcoding of *16S rDNA* and *COI* genes. BLAST analysis revealed 98.9–100% identity of two ticks, one identified for both genes, with *H. aegyptium* (GenBank accession numbers MG418679, AF132821 and KY548846). Phylogenetic analysis was performed to evaluate the genetic association between the sequenced samples and other *Hyalomma* species obtained from the GenBank database (Figs. 3 and 4). Both phylogenetic trees present clusters of the genotypes *H. marginatum*, *H. excavatum*, *H. aegyptium* and *H. impeltatum* and an outgroup of *Ixodes ricinus* (GenBank accession number MH645522 and MZ305543). The samples retrieved in this study cluster in the subgroup of *H. aegyptium*, being aggregated with samples collected from Turkey (KR870970) or Israel (KU130407), in the case of *16S rDNA* sequences, and from Israel (KT989617), Morocco (OL467652) or Algeria (OL467646) in *COI* sequences.

Sequence and BLAST analysis of suspected positive samples revealed four tick pools as positive for the *Ehrlichia 16S rRNA* gene (7.84%), and one (1.96%) as positive for the *Rickettsia ompA* gene. BLAST analysis of the *Ehrlichia 16S rRNA* gene of *H. aegyptium* showed three samples sharing 98–99% identity with *Candidatus* M. mitochondrii (GenBank accession number MG668797, OQ320500 or MK416236.1) and one with 99.6% identity to *Ehrlichia ewingii* (GenBank accession number MW092750). One sample (isolate 12) positive to *Ehrlichia 16S rRNA* presented a co-infection with *Rickettsia* sharing 99.7% identity with *Rickettsia africae* when targeting the *ompA* gene (GenBank accession number MW874463).

**Fig. 5:**
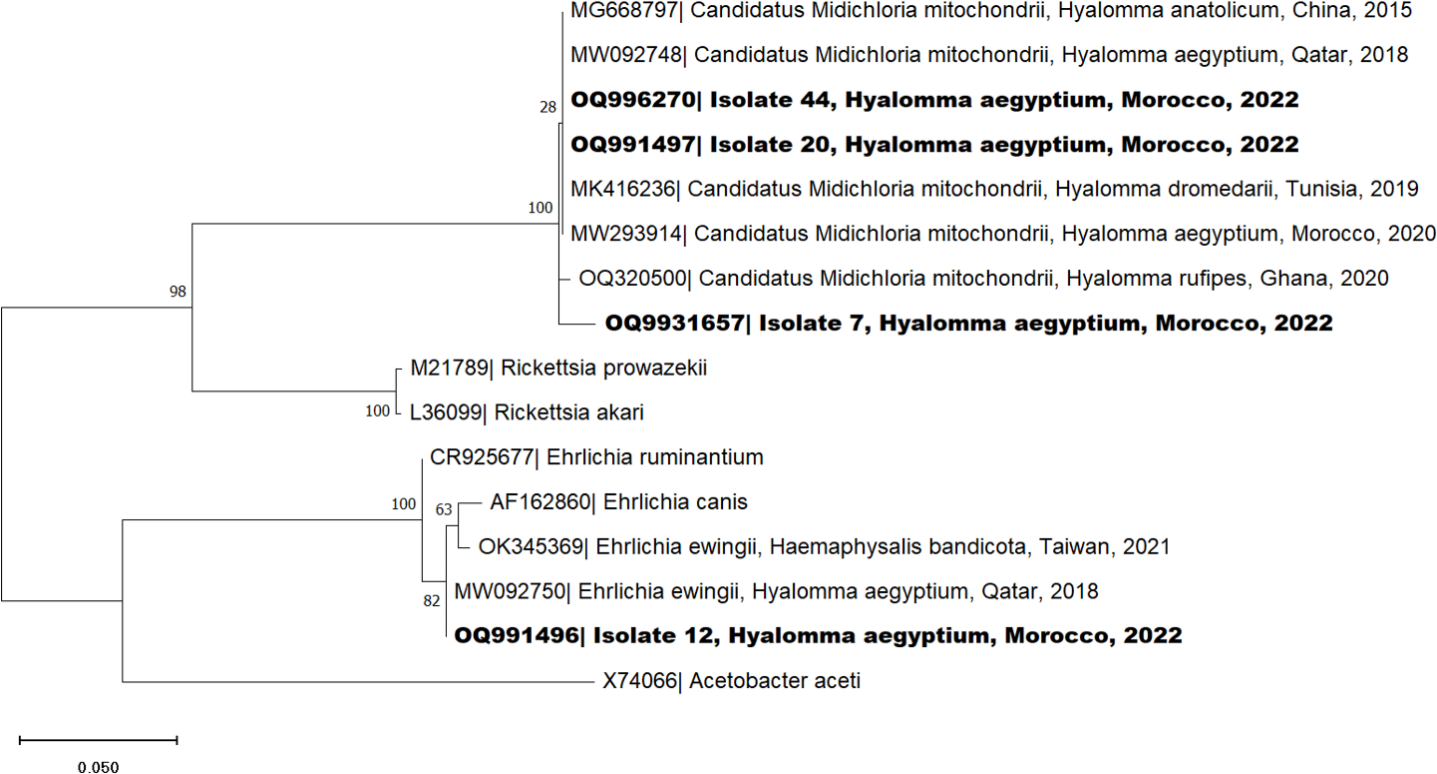
Phylogenetic tree of *Ehrlichia* (*16S rRNA*) sequences of *Hyalomma aegyptium* isolated from spur-thighed tortoise (*Testudo graeca*), Morocco. See Fig. 3 for details of the analysis

**Fig. 6:**
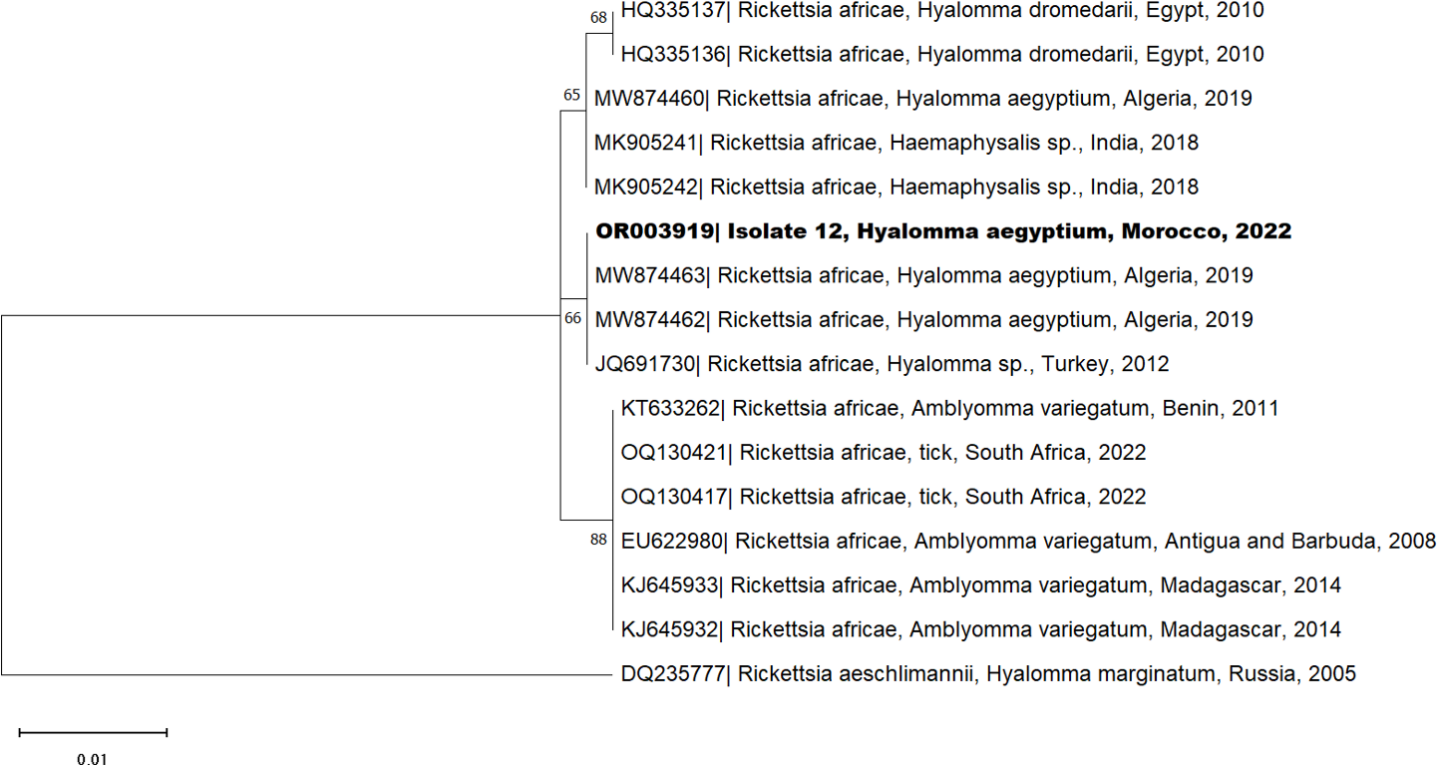
Phylogenetic tree of *Rickettsia* (*ompA*) sequences of *Hyalomma aegyptium* isolated from spur-thighed tortoise (*Testudo graeca)*, Morocco. See Fig. 3 for details of the analysis

Phylogenetic analysis for the *Ehrlichia 16S rRNA* (Fig. 5) confirmed the classification as *Candidatus* M. mitochondrii and *E. ewingii*. It shows a cluster between the isolates 44 (OQ996270), 20 (OQ991497) and 7 (OQ9931657) and *Candidatus* M. mitochondrii detected in *H. anatolicum* ticks from China (MG668797), *H. aegyptium* from Qatar (MW092748) and Morocco (MW293914), *H. dromedarii* from Tunisia (MK416236) and *H. rufipes* from Ghana (OQ320500). Concerning isolate 12 (OQ991496), it clusters with sequences identified as *E. ewingii* collected from *Haemaphysalis bandicota* from Taiwan (OK345369) and *H. aegyptium* from Qatar (MW092750). In terms of the *Rickettsia ompA* sequences, the phylogenetic analysis confirms the classification as *R. africae* (Fig. 6). The positive sample (isolate 12 - OR003919) clusters with *R. africae* sequences from Turkey (JQ691730) or Algeria (MW874462). However, tick samples were PCR-negative for *Babesia* spp., *Borrelia* spp., *C. burnetii*, CCHFV, and *Hepatozoon* spp. / *H. mauritanica* infectious agents.

The infectious agents search in tortoises’ blood, which included *Anaplasma* spp., *Ehrlichia* spp. and *Rickettsia* spp., detected no positive samples.

## Discussion

Assessing the impact of *H. aegyptium* infestation intensity on the spur-thighed tortoise’s health status and the potential transmission of zoonotic infectious agents are crucial aspects to improve conservation strategies for this vulnerable species and promote human health (Laghzaoui et al. 2022). Our study documents high prevalence and medium infestation intensity of *H. aegyptium* in spur thigh-tortoise, and the influence of tortoise sex and female reproductive stage in the infestation rate. The *H. aegyptium* ticks exhibited a minimum infection rate, calculated as the number of positive pools to the total number of ticks tested, of 0.61–1.84% of infectious agents, harbouring *R. africae*, *Candidatus* M. mitochondrii and *E. ewingii* species. The lack of transference of those agents to the spur-thighed tortoise imposes a greater concern for human health problems, primarily due to high human contact through collecting them as pets (Segura et al. 2020; Segura and Acevedo 2019), rather than posing significant health and demographic problems for the tortoises themselves.

The tick prevalence of the Mediterranean spur-thighed tortoises in the Maamora forest has been documented to be one of the highest in their distribution range (Gharbi et al. 2015; Tiar et al. 2016; Najjar et al. 2020; Table S2). Acknowledging that this is a 1-year study, it shows medium infestation intensity, when compared to other studies (e.g., Robbins et al. 1998; Brianti et al. 2010; Gharbi et al. 2015; Tiar et al. 2016), and allows comparisons with the previous study of 2018. Segura et al. (2019) detected higher infestation intensity, which might be associated with the decrease of temperatures and humidity in 2022 (134 mm and a minimum temperature of 8.7 ºC in the spring and winter of 2018 and 56 mm and 10 ºC in the spring and winter of 2022; Meterological station Tiflet). Temperature and humidity are crucial determinants for the distribution and development of ticks, which limits their abundance and distribution (Javanbakht et al. 2015). Overall, in this Mediterranean forest, the high tick prevalence and medium infestation intensity might be the result of the highly dense tortoise population (Segura et al. 2019), which might be interpreted as a host adaptation to the impact of parasites. Ticks make an oriented choice to gather in the most profitable plots (e.g., Barbault 1992), represented in our case by dense host population, as occurred in an Algeria population (Tiar et al. 2016).

In our study, tortoise sex plays a role in tick infestation, with male tortoises presenting higher infestation rates than females, as occurred in our previous study (Segura et al. 2019) and in other populations (Laghzaoui et al. 2022), which could be related to home range differences between sexes (Robbins et al. 1998). Male body condition decreased with higher infestation rates, as reported in 2018 (Segura et al. 2019), which might suppose an extra biological cost. Nevertheless, there was no relationship between tick infestation and the body condition of gravid females. Indeed gravid females presented higher infestation rates compared to non-gravid females, as documented in western lizards (Pollock et al. 2012). This could be caused by nesting search by gravid females, which might increase their home range and therefore the encounter rate of ticks (Tiar et al. 2016). Those facts might affect demographic traits, under conditions where there is not enough energy to support both the immune and reproductive systems (e.g., Hurd 2001; Pollock et al. 2012; Lockley et al. 2020). Indeed, this population has been documented as highly female biased (Segura and Acevedo 2019), and the infestation rate in males may be a factor among others contributing to keep males in low densities. However, tortoise reproductive traits are strongly influenced by other factors such as female age or drought periods. For instance older females produce more and larger clutches (Díaz-Paniagua et al. 2001; Segura et al. 2021) and drought periods strongly reduce female reproduction investment (Rodríguez-Caro et al. 2021). Due to this, further studies coping with long-term data on reproductive females and accounting for environmental variables are crucial for determining the role of tick infestation in reproductive success.

Ticks of *H. aegyptium* carry and transmit several pathogens (Paștiu et al. 2012; Kautman et al. 2016; Barradas et al. 2020; Manoj et al. 2021; Norte et al. 2021). In this study, we detected three species of pathogens, *R. africae*, *E. ewingii*, and *Candidatus* M. mitochondrii, that have been previously detected in spur-thighed tortoise ticks in Morocco (Norte et al. 2021), Qatar (in imported tortoises from pet trade; Barradas et al. 2019, 2020), Israel (Mumcuoglu et al. 2022), and Italy (Manoj et al. 2021). In Africa, the estimated prevalence of *R. africae* in *Hyalomma* ticks is 13.9% (Cossu et al. 2023), and particularly in the North of Morocco, *R. africae* and *Candidatus* M. mitochondrii in spur-thighed tortoises have been reported to present a higher prevalence (2.94 and 14.58%, respectively; Norte et al. 2021) than the one encountered by this study. Additionally, although *Anaplasma* spp., *C. burnetii*, *Babesia* spp., CCHFV and *H. mauritanica* have been documented in other populations of spur-thighed tortoises infested by *H. aegyptium* throughout their distribution range (Paștiu et al. 2012; Kautman et al. 2016; Akveran et al. 2020; Mumcuoglu et al. 2022; Rjeibi et al. 2022), our study did not yield positive results. Indeed, for example, Africa presents a low estimated prevalence of *C. burnetii* (Cossu et al. 2023), and in Morocco, of the four pathogens, only *H. mauritanica* has been detected in spur-thighed tortoises, with a low prevalence of 0–2.1%, being higher in eastern regions (Široký et al. 2009; Norte et al. 2021). The low prevalence of pathogens in ticks might be related to the range and abundance of other potential hosts (wildlife, livestock, or domestic animals), host predation, barriers within ticks – e.g. their immune system potentially influences their infection –, potential co-infection with other pathogens impacting ticks and their ability to maintain the infection and/or possibly infect hosts, and environmental variables including temperature, humidity, daylight duration, and season (Daniel et al. 1976; Randolph 2004; de la Fuente et al. 2017).

The pathogens encountered in *H. aegyptium* may impact both domestic and wild animal health, causing, e.g., granulocytic anaplasmosis, ehrlichiosis, or coxiellosis (Wernery, 2014). Some of these diseases lead to asymptomatic (e.g., CCHFV; Temur et al. 2021) or non-specific symptoms such as fever (e.g., *Anaplasma* spp. or *Ehrlichia* spp.; Karlsen et al. 2020), whereas others lead to reproductive losses like abortions, stillbirths or weak offspring in wild mammals and birds, among others (e.g., *C. burnetii*; González-Barrio and Ruiz-Fons 2019; Celina and Cerný 2022). However, acknowledging the limited study of the effects of such pathogens in reptiles, it results in anaemia, dehydration or emaciation (Mendoza-Roldan et al. 2021), symptoms which might be overlooked or associated with other factors. Accordingly, the absence of pathogens in the tortoise blood samples suggests that the infection of ticks occurred from another source other than the spur-thighed tortoises or that the transmission of pathogens from vector to host was inefficient (Rocha et al. 2022). Previous studies have successfully detected pathogens in blood, demonstrating that spur-thighed tortoises could serve as reservoirs and/or sources of tick-borne infections (Akveran et al. 2020; Kar et al. 2020; Mihalca et al. 2008; Široký et al. 2009). The effect of pathogens on the tortoise’s health, although seldom reported, seems to be minimal or even inexistent (Mihalca et al. 2008), pinpointing the coevolution of tortoises as a host species according to the long-term exposure. On the other hand, pathogens found in the ticks attached to spur-thighed tortoises might affect human health due to the ability of *H. aegyptium* to feed on humans (Vatansever et al. 2008) and the high collection of this tortoise species as a pet throughout their whole distribution (Segura et al. 2020). Both *E. ewingii* and *R. africae* are zoonotic pathogens inducing, respectively, monocytic ehrlichiosis (Andoh et al. 2015) and African tick bite fever – a systemic fever in travellers from Africa (Jensenius et al. 2004). Therefore, the study raises concern about the collection of spur-thighed tortoises as pets due to the emerging or re-emerging of zoonotic infections.

Spur-thighed tortoise management and conservation programs might include long-term studies to determine the tortoise epidemiological status and the transmission of zoonotic infectious agents, accounting for both, demographic drivers (sex, age, reproduction) and abiotic drivers (temperature, rainfall, vegetation cover) that affect the tick infestation in the host.

### Electronic supplementary material

Below is the link to the electronic supplementary material.

Supplementary Material 1: **Supplementary Table S1**. Data from sampled tortoises, their morphologic characteristics and identification of PCR-positive results. **Supplementary Table S2.** Tick infestation in *Testudo graeca* populations through their distribution range: infestation prevalence (%), infestation intensity (tick/infected tortoise) and host density (tortoise/ha)
