# Supplementary material for: Tick infestation in spur-thighed tortoise population: a pilot study for unraveling epidemiological patterns and demographic consequences
Source: Exp Appl Acarol. 2023 Nov 16;91(4):661–79. doi: 10.1007/s10493-023-00863-7 (PMC10689538; doi:10.1007/s10493-023-00863-7)
Supplement: Supplementary file 1 — Supplementary Material 1: Supplementary Table S1. Data from sampled tortoises, their morphologic characteristics and identification of PCR-positive results. Supplementary Table S2. Tick infestation in Testudo graeca populations through their distribution range: infestation prevalence (%), infestation intensity (tick/infected tortoise) and host density (tortoise/ha) [file 10493_2023_863_MOESM1_ESM.docx]

**Supplementary information**

**Tick Infestation in Spur-thighed Tortoise Population: Unraveling Epidemiological Patterns and Demographic Consequences**

Amalia Segura^1&*^, Marta Rafael^2&^, Rita Vaz-Rodrigues^2^, Oscar Rodríguez^1^, Christian Gortázar^2^, José de la Fuente^2,3^

^1^BP 30, Sidi Allal el Bahraoui 15250, Morocco

^2^ SaBio, Instituto de Investigación en Recursos Cinegéticos (IREC), Consejo Superior de Investigaciones Científicas (CSIC), Universidad de Castilla-La Mancha (UCLM)-Junta de Comunidades de Castilla-La Mancha (JCCM), Ronda de Toledo 12, 13005 Ciudad Real, Spain

^3^ Center for Veterinary Health Sciences, Department of Veterinary Pathobiology, Oklahoma State University, Stillwater, OK 74078, USA

^&^Equal contribution

**Table S1.** Data from sampled tortoises, their morphologic characteristics and identification of PCR-positive results.

| Turtle ID | Gender | Age (years) | Body condition | Carapace length (mm) | Weight (gr) | Number of ticks | PCR-positive pathogens |
| --- | --- | --- | --- | --- | --- | --- | --- |
| 3 | F | 30 | -95.2 | 195 | 1223 | 5 |  |
| 4 | M | 15 | -83.3 | 132 | 463 | 3 |  |
| 5 | M | 16 | -139.7 | 162 | 690 | 11 |  |
| 7 | F | 30 | -205.1 | 193 | 1088 | 4 |  |
| 9 | F | 29 | -90.5 | 199 | 1278 | 3 |  |
| 20 | F | 35 | -262.5 | 199 | 1106 | 2 |  |
| 22 | M | 21 | 69.7 | 150 | 786 | 1 |  |
| 32 | F | 17 | -92.4 | 172 | 937 | 2 |  |
| 38 | F | 19 | -32.3 | 195 | 1286 | 4 |  |
| 40 | F | 22 | -117.9 | 182 | 1037 | 4 |  |
| 45 | M | 28 | -115.2 | 152 | 620 | 12 |  |
| 50 | F | 16 | -2.27 | 195 | 1316 | 1 |  |
| 52 | M | 23 | -57.6 | 155 | 706 | 6 |  |
| 73 | F | 19 | -163.1 | 168 | 816 | 4 |  |
| 83 | F | 19 | -8.27 | 195 | 1310 | 1 | *Candidatus* Midichloria mitochondrii |
| 92 | M | 20 | -104.9 | 149 | 602 | 7 |  |
| 93 | F | 21 | -18.1 | 193 | 1275 | 3 |  |
| 97 | F | 25 | 64.9 | 200 | 1446 | 3 |  |
| 100 | F | 19 | 138.1 | 173 | 1180 | 3 |  |
| 103 | F | 26 | -175.9 | 205 | 1268 | 10 |  |
| 107 | F | 19 | -13.2 | 186 | 1192 | 7 |  |
| 109 | F | 30 | 360.3 | 194 | 1666 | 8 |  |
| 123 | F | 20 | 25.9 | 175 | 1093 | 2 |  |
| 131 | F | 15 | 14.6 | 131 | 529 | 2 |  |
| 173 | F | 20 | -160.5 | 165 | 781 | 4 |  |
| 179 | F | 22 | -183.1 | 175 | 884 | 4 |  |
| 191 | F | 20 | 84.1 | 155 | 900 | 2 |  |
| 206 | F | 21 | -1.9 | 178 | 1103 | 5 |  |
| 212 | F | 16 | -12.9 | 182 | 1142 | 4 |  |
| 222 | F | 15 | -85.9 | 132 | 441 | 3 |  |
| 223 | F | 20 | -63 | 175 | 1004 | 2 |  |
| 230 | F | 22 | -119.4 | 181 | 1023 | 6 |  |
| 231 | M | 20 | 166.7 | 161 | 987 | 2 |  |
| 232 | F | 23 | 49.5 | 190 | 1305 | 4 |  |
| 233 | F | 28 | 56.7 | 195 | 1375 | 5 | *Rickettsia africae* and *Ehrlichia ewingii* |
| 234 | F | 20 | -111.6 | 151 | 654 | 3 |  |
| 235 | M | 33 | 96.8 | 152 | 832 | 3 |  |
| 236 | F | 15 | -81.4 | 140 | 546 | 8 |  |
| 237 | F | 20 | 63.1 | 180 | 1193 | 8 |  |
| 238 | M | 23 | -42.6 | 155 | 721 | 7 |  |
| 239 | M | 19 | 110.7 | 134 | 676 | 3 |  |
| 240 | F | 25 | -85.3 | 179 | 1032 | 3 |  |
| 246 | M | 23 | 33.6 | 150 | 750 | 2 |  |
| 247 | F | 25 | 207.3 | 185 | 1400 | 7 |  |
| 248 | F | 23 | -28.4 | 147 | 687 | 3 |  |
| 250 | M | 20 | 131.6 | 132 | 678 | 1 |  |
| 251 | M | 19 | -22.2 | 143 | 628 | 10 |  |
| 254 | M | 24 | -5.6 | 155 | 758 | 6 |  |
| 257 | M | 19 | 35.6 | 141 | 667 | 6 | *Candidatus* Midichloria mitochondrii |
| 276 | F | 16 | 32.2 | 171 | 1049 | 8 |  |
| 280 | F | 25 | -121.7 | 185 | 1071 | 4 |  |
| 304 | F | 18 | 55.3 | 185 | 1248 | 2 |  |
| 310 | F | 20 | 93.9 | 168 | 1073 | 5 |  |
| 311 | M | 18 | -127.8 | 151 | 598 | 10 |  |
| 315 | M | 18 | 63.6 | 141 | 695 | 2 |  |
| 316 | M | 20 | -118.3 | 159 | 683 | 11 |  |
| 321 | F | 23 | 247.2 | 171 | 1264 | 4 |  |
| 324 | F | 22 | 155.8 | 202 | 1562 | 1 |  |
| 333 | F | 19 | -13.8 | 180 | 1116 | 5 |  |
| 351 | F | 23 | 74.3 | 153 | 865 | 4 |  |
| 352 | F | 17 | 26.9 | 159 | 893 | 4 |  |
| 355 | M | 20 | 102.7 | 134 | 668 | 4 |  |
| 356 | M | 19 | 86.8 | 161 | 907 | 4 |  |
| 358 | F | 19 | -106.1 | 193 | 1187 | 2 |  |
| 360 | F | 25 | -40.8 | 205 | 1403 | 4 |  |
| 362 | F | 24 | 84.8 | 202 | 1491 | 9 |  |
| 363 | F | 20 | 46.3 | 185 | 1239 | 7 |  |
| 364 | F | 20 | -172.9 | 198 | 1183 | 4 |  |
| 365 | F | 25 | 106.4 | 181 | 1036 | 4 |  |
| 366 | F | 19 | -16.1 | 193 | 1277 | 4 |  |
| 367 | F | 30 | 125.2 | 212 | 1657 | 3 |  |
| 368 | F | 21 | 28.8 | 193 | 1322 | 3 |  |
| 369 | F | 14 | 7.7 | 195 | 1326 | 4 |  |
| 370 | F | 18 | -52 | 200 | 1329 | 2 |  |
| 374 | M | 16 | 112.2 | 160 | 923 | 4 |  |
| 381 | F | 21 | -32.5 | 190 | 1223 | 2 |  |
| 382 | F | 25 | -149 | 200 | 1232 | 4 |  |
| 383 | F | 19 | -48.1 | 193 | 1245 | 2 |  |
| 384 | F | 19 | -24.7 | 185 | 1168 | 4 |  |
| 385 | F | 30 | -33.4 | 190 | 1222 | 3 |  |
| 386 | F | 19 | 30.9 | 175 | 1098 | 2 |  |
| 387 | F | 25 | -20.7 | 185 | 1172 | 1 |  |
| 388 | F | 17 | -134.9 | 155 | 681 | 3 |  |
| 404 | M | 17 | -30.8 | 160 | 780 | 4 |  |
| 410 | F | 23 | 23.5 | 178 | 1128 | 3 |  |
| 411 | F | 19 | 54.9 | 200 | 1436 | 6 |  |
| 412 | F | 22 | 80.5 | 190 | 1336 | 3 |  |
| 413 | F | 9 | 46.3 | 185 | 1239 | 2 |  |
| 414 | M | 16 | 76.8 | 161 | 897 | 7 |  |
| 415 | F | 15 | -79.8 | 180 | 1050 | 2 |  |
| 416 | F | 17 | -100.5 | 165 | 841 | 1 |  |
| 417 | F | 15 | 16.3 | 185 | 1209 | 1 |  |
| 418 | F | 20 | -11.5 | 199 | 1357 | 4 |  |
| 419 | F | 16 | -34.9 | 173 | 1007 | 1 |  |
| 420 | M | 15 | -70.3 | 141 | 561 | 5 |  |
| 421 | M | 19 | -20.3 | 150 | 696 | 3 |  |
| 422 | F | 823 | 24.5 | 190 | 1280 | 1 |  |
| 423 | F | 21 | 1.86 | 178 | 1291 | 4 |  |
| 424 | F | 24 | 38.8 | 193 | 1332 | 2 |  |
| 425 | M | 23 | -108.5 | 130 | 419 | 7 |  |
| 426 | F | 25 | 36.1 | 205 | 1480 | 5 |  |
| 427 | M | 15 | 24.6 | 141 | 656 | 3 |  |
| 430 | F | 20 | -135.3 | 195 | 1183 | 4 |  |
| 431 | F | 23 | -104.1 | 175 | 963 | 3 |  |
| 434 | F | 21 | -97.4 | 172 | 932 | 3 |  |
| 435 | M | 19 | -94.3 | 150 | 622 | 4 | *Candidatus* Midichloria mitochondrii |
| 436 | F | 20 | 62.3 | 185 | 1255 | 2 |  |
| 437 | M | 15 | 77.2 | 160 | 888 | 3 |  |
| 440 | M | 20 | -1.3 | 141 | 630 | 2 |  |
| 450 | F | 17 | 12.5 | 140 | 640 | 3 |  |
| 452 | F | 19 | 178.5 | 199 | 1547 | 3 |  |
| 454 | F | 17 | 153.3 | 169 | 1145 | 1 |  |
| 455 | F | 25 | 227.6 | 188 | 1458 | 2 |  |
| 456 | F | 21 | 68.6 | 181 | 1211 | 4 |  |
| 457 | F | 19 | 140.2 | 171 | 1157 | 2 |  |
| 458 | F | 21 | 28.1 | 180 | 1158 | 8 |  |
| 459 | F | 21 | 236.2 | 171 | 1253 | 4 |  |
| 460 | F | 18 | 84.1 | 155 | 900 | 1 |  |
| 461 | F | 15 | 84.5 | 149 | 825 | 2 |  |
| 463 | F | 21 | 123.9 | 175 | 1191 | 2 |  |
| 469 | F | 20 | -65.3 | 195 | 1253 | 9 |  |
| 470 | F | 17 | 73.5 | 158 | 927 | 3 |  |
| 471 | F | 20 | 43.5 | 190 | 1299 | 1 |  |
| 472 | M | 17 | -61.9 | 140 | 560 | 8 |  |
| 473 | F | 20 | 30.5 | 190 | 1286 | 4 |  |
| 474 | F | 17 | -33.6 | 194 | 1270 | 4 |  |
| 475 | F | 25 | 69.2 | 196 | 1400 | 3 |  |
| 476 | M | 16 | 15.8 | 145 | 685 | 4 |  |
| 477 | F | 18 | -146.3 | 163 | 770 | 4 |  |
| 499 | F | 25 | -103.4 | 197 | 1240 | 5 |  |

**Table S2.** Tick infestation in Testudo graeca populations through their distribution range: infestation prevalence (%), infestation intensity (tick/infected tortoise) and host density (tortoise/ha)**.**

| Country | Region | Host  density | Sample  size | Infestation  prevalence | Infestation  intensity | Reference |
| --- | --- | --- | --- | --- | --- | --- |
| Africa | North Africa | - | 585 | 37.8 | 3.9 | (Brianti et al., 2010) |
| Algeria | El Kala | 4.4 | 65 | 85 | 4.6 | (Tiar et al., 2016) |
| Algeria | Aflou | 5.8 | 60 | 90 | 5.9 | (Tiar et al., 2016) |
| Algeria | Djelfa | 11.2 | 41 | 100 | 9.4 | (Tiar et al., 2016) |
| Algeria | Laghouat | 1.6 | 35 | 9 | 1.7 | (Tiar et al., 2016) |
| Morocco | Souss Valley | - | 29 | 20.7 | - | (Široký et al., 2009) |
| Morocco | Maamora | - | 23 | 43.5 | 1.5 | [(Laghzaoui et al., 2022)](https://www.tandfonline.com/author/Laghzaoui%2C+El-Mustapha) |
| Morocco | Had Draa |  | 10 | 100 | 12 | (Laghzaoui et al., 2022) |
| Morocco | Central Jbilet Mountains |  | 193 | 81.4 | 7.99 | (Laghzaoui et al., 2022) |
| Morocco | Oued Zat |  | 40 | 95 | 3.97 | (Laghzaoui et al., 2022) |
| Morocco | Sidi Kaouki |  | 105 | 71.4 | 7.81 | (Laghzaoui et al., 2022) |
| Morocco | Timnoutine |  | 11 | 100 | 9.36 | (Laghzaoui et al., 2022) |
| Morocco | Maamora | 26.5 | 296 | 92.5 | 6.7 | (Segura et al., 2019) |
| Tunisia |  | - | 210 | 66.2 | 4.3 | (Gharbi et al., 2015) |
| Turkey |  | - | 26 | 96.2 | 10.4 | (Akveran et al., 2020) |
